# Validation of the Ontario Protocol Assessment Level (OPAL) Tool for Assessing Clinical Trial Complexity and Supporting Workforce Planning in the Italian Clinical Research Context

**DOI:** 10.1007/s43441-026-01006-x

**Published:** 2026-06-30

**Authors:** Daniele Napolitano, Mattia Bozzetti, Rosario Caruso, Monica Guberti, Ileana Frau, Bobbi Smuck, Gionata Fiorino, Franco Scaldaferri, Lucrezia Laterza, Antonio Gasbarrini, Vincenzina Mora

**Affiliations:** 1 Scientific Direction – Fondazione Policlinico Universitario IRCCS, Università Cattolica del Sacro Cuore Rome, Largo A. Gemelli 1, 00168 Rome, Italy; 2 Department of Biomedicine and Prevention, University of Rome Tor Vergata, Rome, Italy; 3 Direction of Health Professions, ASST Cremona, Cremona, Italy; 4 Department of Biomedical Sciences for Health, University of Milan, Milan, Italy; 5 Health Professions Research and Evidence Transfer Unit, IRCCS MultiMedica, Sesto San Giovanni, Italy; 6 Nursing and Allied Health Professions, IRCCS Istituto Ortopedico Rizzoli, Bologna, Italy; 7 Ass. Prime Site Director, Strategic Site Solutions EMEA - IQVIA, Milan, Italy; 8 Department of Ophthalmology, Ivey Eye Institute, St. Joseph’s Health Care, London, ON Canada; 9 Gastroenterology and Digestive Endoscopy, San Camillo-Forlanini Hospital, Rome, Italy; 10 Centro Malattie dell’Apparato Digerente (CEMAD), Fondazione Policlinico Universitario Agostino Gemelli, IRCCS, Università Cattolica del Sacro Cuore, Rome, Italy

**Keywords:** Clinical trial complexity, Workload assessment, Feasibility analysis, Clinical research workforce, Regulatory science, Trial operations

## Abstract

**Introduction:**

The growing complexity of clinical trials, particularly in oncology, has significantly increased the operational burden on research staff. However, standardized and validated instruments to measure trial-related workload remain scarce. This study aimed to adapt and validate the Italian version of Ontario Protocol Assessment Level (I-OPAL) tool for the Italian context, providing a reliable framework for workload planning and feasibility assessment.

**Methods:**

A cross-sectional, multicenter study was conducted across Italian research institutions. The OPAL tool was translated and culturally adapted following established validation procedures. Content validity was assessed using the Content Validity Ratio (CVR), while inter-rater reliability was evaluated with the intraclass correlation coefficient (ICC). Discriminant validity was examined through non-parametric tests, effect size measures, and multiple correspondence analysis.

**Results:**

A total of 513 clinical trials were included. The OPAL tool showed excellent inter-rater reliability (ICC = 0.93) and all items met the minimum CVR threshold (0.78–1.00). Significant differences in OPAL scores were observed across study type, clinical setting, sample size, and duration (all *p* < 0.001), with large effect sizes (ε^2^ up to 0.645). Higher OPAL scores were positively correlated with greater allocation of research staff, particularly nurses and data managers. Sensitivity analyses confirmed the robustness of findings, and internal consistency checks revealed full alignment with the model’s classification rules.

**Conclusions:**

The Italian validation of OPAL confirmed its reliability, validity, and practical relevance as a tool for standardized workload assessment in clinical research. Its integration into feasibility analyses and trial planning could enhance resource allocation, regulatory compliance, and sustainability of research activities in Italy.

## Introduction

In recent years, the landscape of clinical trials has undergone a marked transformation, characterized by a steady increase in protocol complexity and operational demands. This evolution is particularly evident in oncology, where contemporary trials frequently involve intricate procedures, extended follow-up schedules, complex eligibility criteria, multiple endpoints, and rapidly evolving regulatory requirements [1–3]. These changes have had a direct impact on the workload of clinical research professionals, including clinical study coordinators (CSCs), data managers, and clinical research nurses (CRNs), whose roles are pivotal to ensuring the quality and integrity of trial conduct [4]. As a consequence, the ability to accurately and consistently measure clinical trial workload has become a strategic priority for research institutions, informing decisions related to staffing, budgeting, feasibility assessment, and risk management. Despite this growing need, workload estimation in clinical research remains largely dependent on subjective judgment or fragmented time-tracking practices, which are insufficient to capture the multifactorial nature of protocol-related complexity [5, 6]. Most research centers still rely on subjective estimates or fragmented documentation of time allocation, which fails to capture the multifactorial nature of trial-related complexity [4].

To address this operational challenge, several international instruments have been developed to assess protocol complexity and its implications for workload. A recent scoping review identified twelve tools designed to support workload assessment in clinical trials, many of which were originally developed within oncology settings [4]. These include the ASCO Clinical Trial Workload Assessment Tool [7], the Research Effort Tracking Application (RETA) [8], the Trial Rating and Complexity Assessment Tool (TRACAT) [9], and the Relative Value of Work (RVW) system [10]. Italy has also contributed two notable models: the IRST Workload Assessment Tool (IWAT) [11], which evaluates CSC workload, and the Nursing Time Required by Clinical Trial Assessment Tool (NTRCT-AT) [6], which focuses on estimating CSC time.

Among these instruments, the Ontario Protocol Assessment Level (OPAL) stands out for its simplicity, applicability, and potential for cross-setting adaptation [12]. Developed by the Ontario Institute for Cancer Research, OPAL is a protocol complexity rating scale that classifies trials using a tiered system based on core and incremental activities. It assigns scores ranging from 1 to 8 (with optional increments up to 10) and accounts for study design, procedures, monitoring requirements, and trial phase [12]. Importantly, OPAL allows for the calculation of protocol workload, case workload (per patient), and total workload, supporting individualized and department-level planning.

What makes OPAL particularly valuable is its proven feasibility and inter-rater reliability in multicenter pilot studies and its flexibility across diverse clinical trial environments. Moreover, it is—so far—the only tool to have undergone cross-cultural adaptation beyond its original context, having been preliminarily validated in Portuguese [13]. However, no validated Italian version currently exists.

In the Italian context, the growing volume and complexity of clinical trials have further highlighted the need for structured approaches to workload assessment. CSCs and CRNs play a central role in trial implementation and coordination; however, their professional profiles and responsibilities are not yet uniformly defined at the national level, resulting in considerable variability across institutions [14–16]. This organizational heterogeneity translates into inconsistent task allocation and limits the use of objective criteria to support staffing and resource planning. These challenges have become even more salient following the implementation of EU Regulation 536/2014, which requires research centers to document adequate infrastructure, staffing, and organizational capacity in formal feasibility assessments. In the absence of validated and standardized workload assessment tools, meeting these regulatory expectations and justifying resource needs to sponsors remains particularly demanding [4]. Against this background, the present study aims to address the need for a standardized approach to workload assessment in Italian clinical research by adapting and validating the OPAL tool. Specifically, the study involves the linguistic and cultural adaptation of OPAL into Italian, the evaluation of its content validity through expert review, the assessment of inter-rater reliability, and the examination of its discriminant capacity across trials of varying complexity. By providing a validated Italian version of OPAL, this work seeks to support evidence-based workload planning, enhance feasibility assessment processes, and promote sustainable staffing models aligned with contemporary European regulatory standards.

## Materials and Methods

### Study Aim, Design, Setting, and Eligibility Criteria

This study aimed to adapt and validate the OPAL tool within the Italian clinical research context. A cross-sectional design was employed, involving multiple Italian research institutions, including IRCCSs, university hospitals, and public healthcare centers. All ongoing and completed clinical trials at participating sites were eligible for inclusion.

### Scoring System

**Fig. 1:**
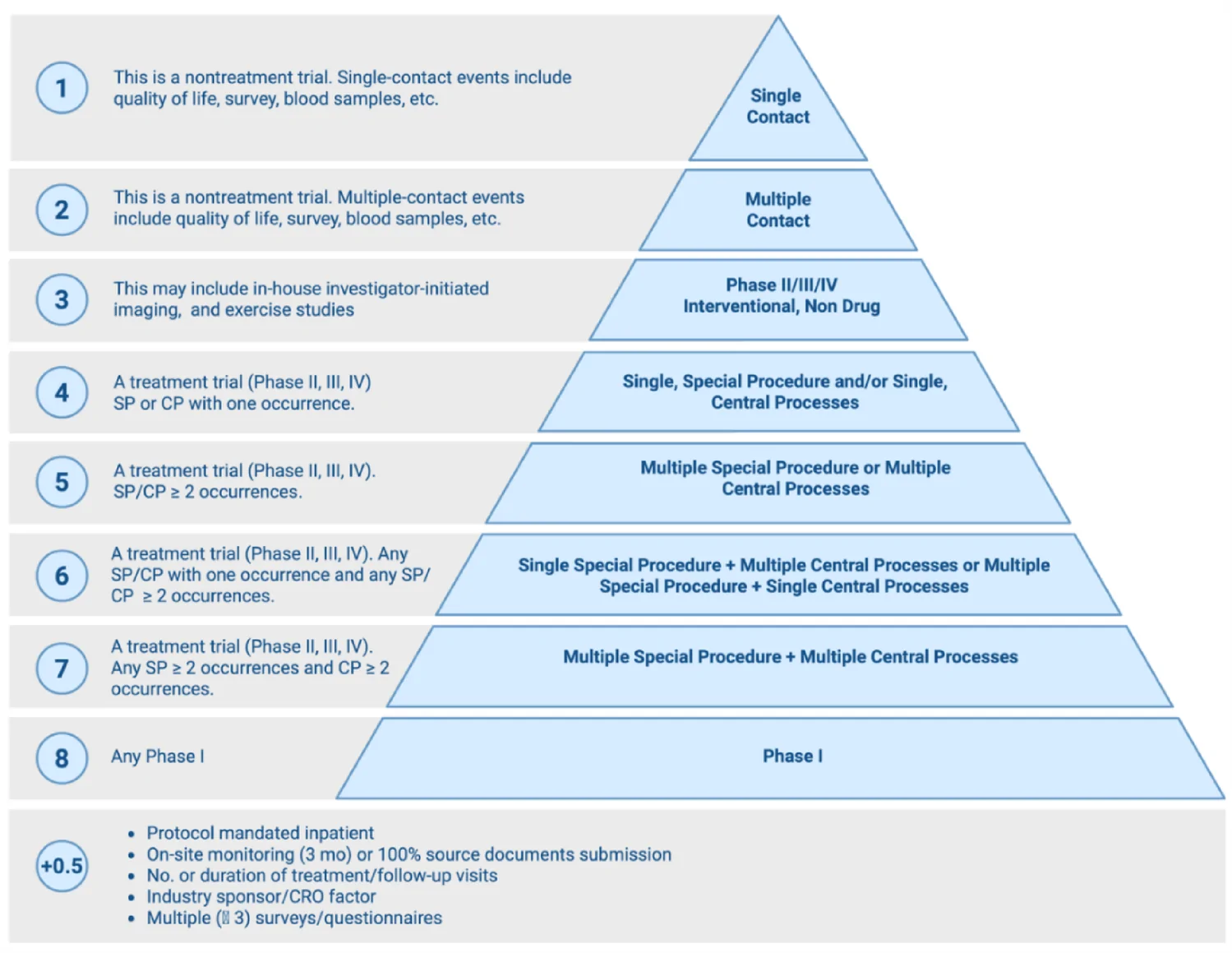
Operational complexity stratification of clinical trials using the OPAL framework. The OPAL model stratifies clinical trials by operational complexity using an 8-level hierarchical structure. Levels 1–3 correspond to non-interventional or minimally interventional studies (e.g., single or multiple contact observational studies), while levels 4–8 represent progressively complex treatment trials, culminating in Phase I studies (level 8). The classification incorporates the presence and frequency of Special Procedures (SP) and Central Processes (CP), with higher levels requiring multiple occurrences of both. Optional modifiers (+ 0.5 each) allow additional granularity for protocol-specific factors such as inpatient requirements, intensive monitoring, industry/CRO involvement, and extensive survey administration, up to a theoretically maximum score of 10.5

The system is based on a pyramid-like scale with *n* = 8 core levels, ranging from low-complexity, non-interventional studies (levels 1–2) to highly complex early-phase interventional trials (level 8) (Fig. 1) [12]. Each ascending level reflects an increase in trial demands, such as workload intensity, data requirements, and regulatory oversight. To ensure practical applicability across a wide variety of study types and disease sites, the model was designed to be intuitive and flexible. Optional modifiers—each contributing an additional 0.5 points—were introduced to capture study-specific features not fully represented by the base levels. These include factors such as extended duration, industrial sponsorship, or complex design formats (e.g., basket or umbrella trials), allowing for a maximum total score of 10.

The OPAL scoring system provides a structured method for quantifying clinical trial workload by integrating protocol complexity with patient management requirements. Table 1 presents the formulas used to estimate different components of trial-related workload, including patient activity and protocol oversight.

**Table 1:** OPAL workload calculation formulas

Component	Formula	Description
Active case workload	Active cases × OPAL score	Full workload per patient actively receiving intervention
Follow-up case workload	Follow-up cases × OPAL score × 0.5	Follow-up cases are weighted at 50%, assuming reduced intensity
Total case workload	(Active cases × OPAL score) + (Follow-up cases × OPAL score × 0.5)	Sum of active and follow-up case workload
Protocol workload	OPAL score	One-time score applied per protocol to reflect management complexity
Total study workload	Total case workload + Protocol workload	Overall workload of the trial, combining case-based and protocol-based burden
Individual staff workload	Σ (Study workload × % Allocation)	Staff-specific workload if responsibilities are shared (e.g., 50% CSC, 50% DM)
Department workload	Σ (All individual workloads)	Total workload across all staff; useful for resource planning and capacity monitoring

OPAL = Ontario protocol assessment level; CSC = clinical study coordinator; DM = data manager

### Validation Process

#### Translation and Adaptation

The translation and cultural adaptation of the OPAL tool into Italian followed a multi-step procedure that combined traditional linguistic validation principles with computational semantic analysis, drawing from the methodological framework proposed by Parozzi et al. [17]. A group of professionals (*n* = 9), including CSCs, DMs and CRNs reviewed the draft to ensure clarity, conceptual equivalence, and clinical relevance. Following expert feedback, minor revisions were made to refine wording and terminology, resulting in the finalized Italian version of the OPAL scoring system, which is available upon request from the corresponding author.

#### Content Validity

Content validation of the OPAL scoring system was assessed using the Content Validity Ratio (CVR) method proposed by Ayre and Scally [18]. A panel of *n* = 9 clinical research professionals (*n* = 3 CSCs, *n* = 2 medical PIs, *n* = 2 DMs, *n* = 1 CRN, and *n* = 1 regulatory affairs specialist) participated in the evaluation, all with at least five years of experience in academic or industry-sponsored trials. Each expert independently rated the items of the OPAL scoring system (8 core levels and 5 optional modifiers) on whether they considered each item “essential” for measuring protocol complexity in clinical trials. For each item, the CVR was computed using the formula: CVR = (n_e_ – *N*/2)/(*N*/2), where n_e_ is the number of panelists indicating the item as essential, and *N* is the total number of panelists.

According to the critical CVR threshold for a panel of 9 raters (minimum acceptable CVR = 0.78), all items met the criteria for content validity. CVR values ranged from 0.78 to 1.00, indicating high agreement among experts regarding the relevance of each component in capturing workload and trial complexity. The number of experts (*n* = 9) was chosen in accordance with Ayre and Scally’s recommendations, which suggest that panels of 8–10 participants ensure adequate validity and feasibility for CVR computation [18]. This sample size also reflected the availability of professionals with diverse roles and expertise across participating centers.

### Variables Collected

The dataset included descriptive information on the clinical department or therapeutic specialty (e.g., oncology, hematology, cardiology), the nature of the treatment pathway (single vs. multiple patient contacts), and the trial phase (from I to IV). Additional variables captured whether the study had a translational orientation (translational vs. purely clinical) and the study design typology (e.g., randomized controlled, basket, platform, or cluster trials). Data were also collected regarding the use of special procedures and protocol-specific operational requirements, such as GCP training, informed consent workflows, adverse event monitoring, complex data handling practices, investigational product or drug management when not fully covered by pharmacy services, and laboratory-related activities including sample storage, shipment, and requisition forms when not performed by dedicated laboratory or biological staff. Furthermore, the presence of centralized processes was recorded, including centralized randomization, data oversight, or trial monitoring systems. Clinical trials were selected consecutively from institutional registries across participating centers to ensure a broad and representative sample. OPAL scoring was independently conducted by trained raters at each site using a standardized scoring manual. In cases of discrepancy, ratings were reviewed and resolved through consensus, ensuring consistency and reproducibility across sites.

### Data Analysis

Data analysis proceeded in multiple stages. Descriptive statistics were first computed for each item, with results reported as means (M) and standard deviations (SD).

Statistical analyses were conducted to evaluate the validity and reliability of the OPAL scoring system. Inter-rater reliability was assessed through the intraclass correlation coefficient (ICC), based on a two-way random-effects model with absolute agreement (ICC_[2,1]_) [19]. This model is appropriate when each subject is rated by a different, random sample of raters, and the focus is on the absolute concordance in scores. ICC values were interpreted following established guidelines, where values above 0.75 indicate good reliability, and values greater than 0.90 reflect excellent agreement. Discriminant validity was examined using non-parametric Kruskal–Wallis tests to evaluate whether OPAL scores differed significantly across categorical study characteristics (e.g., type, setting, enrollment, duration). Where appropriate, effect sizes were quantified using Epsilon squared (ε^2^). For binary comparisons (e.g., industry vs. academic sponsor, presence vs. absence of centralized procedures), independent-samples *t*-tests were conducted, accompanied by Cohen’s d to quantify standardized mean differences. In cases of unbalanced sample sizes, non-parametric robustness checks were performed using Cliff’s Delta.

To further explore the multidimensional structure underlying OPAL classifications, Multiple Correspondence Analysis (MCA) was employed. This technique facilitated the visualization of associations between OPAL levels and design components such as centralized processes and study purpose (e.g., translational). A mosaic plot was also generated to assess the independence of categorical variables and highlight patterns of over- or under-representation. Lastly, a sensitivity analysis was performed by recalculating OPAL scores without the optional modifiers, testing the stability of associations with key structural variables. All analyses were conducted in R 4.5.0 [20].

## Results

Table 2 summarizes the characteristics of the 513 included CTs. Most trials were conducted in oncology (47%) or gastroenterology (17%), and nearly half were in advanced clinical phases (Phase III: 47%). The large majority were interventional (93%) and industry-sponsored (92%). Centralized processes and Standard Operating Procedure (SOP) were present in over 95% of protocols, and approximately half (51%) incorporated a translational design component.

**Table 2:** CTs characteristics (*n* = 513)

Variable	Category	*n* (%)
Study	Randomized controlled trial	221 (43.1%)
	Prospective observational (e.g., cohort)	158 (30.8%)
	Non-interventional (e.g., survey, single/multiple)	33 (6.4%)
	Descriptive/cross-sectional	10 (1.9%)
	Platform/basket/umbrella	6 (1.2%)
	Other	85 (16.6%)
Setting	Oncology	242 (47.2%)
	Gastroenterology	89 (17.4%)
	Obstetrics and gynecology	68 (13.3%)
	Cardiology	34 (6.6%)
	Rheumatology	30 (5.8%)
	Other specialties	47 (9.2%)
	Missing/unspecified	3 (0.6%)
Treatment	Interventional	478 (93.2%)
	Non-treatment (survey type)	33 (6.4%)
	Missing	2 (0.4%)
Phase	Phase I	51 (9.9%)
	Phase II–IIa	140 (27.3%)
	Phase IIb–III–IIIa	240 (46.8%)
	Phase IV	21 (4.1%)
	Not applicable	61 (11.9%)
Sponsor	Yes	470 (91.6%)
	No	41 (8.0%)
CRO involvement	Yes	462 (90.1%)
	No	50 (9.8%)
	Missing	1 (0.2%)
Translational design	Yes	260 (50.7%)
	No	250 (48.7%)
	Missing	3 (0.6%)
SOP presence	Yes	492 (95.9%)
	No/Unspecified	21 (4.1%)
Centralized process (CP)	Yes	491 (95.7%)
	No	20 (3.9%)

CRO, Contract research organization; SOP, standard operating procedure; Note: Percentages may not coincide to 100 due to missingness or rounding.

### Inter-Rater Reliability

Inter-rater reliability of the OPAL scoring system was assessed in a random subsample (*n* = 40) of clinical trials independently evaluated by two trained reviewers. The ICC indicated excellent agreement between raters (ICC = 0.93; 95%CI 0.76–0.97).

### Discriminant Validity

Discriminant validity was assessed by examining differences in OPAL scores across study characteristics (Table 3). Significant differences in OPAL scores were observed across all tested variables. In particular, OPAL scores varied substantially by study type (*χ*^2^_(13)_ = 334.96, *p* < 0.001, ε^2^ = 0.645), setting (*χ*^2^_(14)_ = 203.2, *p* < 0.001, ε^2^ = 0.380), and number of enrolled subjects (*χ*^2^_(7)_ = 77.27, *p* < 0.001, ε^2^ = 0.139). Duration of the study also had a small-to-medium effect on OPAL scores (*χ*^2^_(5)_ = 31.95, *p* < 0.001, ε^2^ = 0.053).

**Table 3:** Discriminant validity across study characteristics [1]

Variable	Kruskal–Wallis χ^2^ (df)	*p*-value	Epsilon^2^	Effect size
Duration	31.95 (5)	< 0.001	0.053	Small–medium
Type of study	334.96 (13)	< 0.001	0.645	Very large
Subjects enrolled	77.27 (7)	< 0.001	0.139	Large
Setting	203.2 (14)	< 0.001	0.380	Very large

Significant pairwise differences in OPAL scores were observed across several study types. RCTs and prospective observational studies consistently exhibited higher OPAL scores compared to non-interventional, descriptive, and cross-sectional designs (*p* < 0.001). Post-hoc comparisons across clinical settings revealed several significant differences in OPAL scores. Oncology trials displayed significantly higher complexity compared to Dermatology (*p* < 0.001), Orthopedics (*p* < 0.001), Neurology (*p* < 0.001), and Gastroenterology (*p* < 0.001). Similarly, studies in the Obstetrics-Gynecology setting showed significantly higher OPAL scores than nearly all other specialties, including Cardiology (*p* < 0.001), Neurology (*p* < 0.001), Rheumatology (*p* < 0.001), and Orthopedics (*p* < 0.001). In contrast, no significant differences were observed between smaller specialties such as Pediatrics, Immunology, or Diagnostic units.

Binary comparisons (Table 4) showed significantly higher scores in studies with industry sponsorship (*t*_(42.4)_ = − 6.05, *p* < 0.001, *d* = 1.53), Contract Research Organization (CRO) involvement (*t*_(52.1)_ = − 8.69, *p* < 0.001, *d* = 2.09), translational design (*t*_(462.4)_ = − 10.20, *p* < 0.001, *d* = 0.90), and centralized procedures (*t*_(20.5)_ = − 14.13, *p* < 0.001, *d* = 3.31). Given the extremely unbalanced sample sizes for CPs, Cliff’s delta (δ = 0.932) was computed as a robust non-parametric estimate, confirming a very large effect.

**Table 4:** Discriminant validity across study characteristics [2]

Variable	Welch t (df)	*p*-value	Cohen’s d	Effect size
Sponsor	− 6.05 (42.4)	< 0.001	− 1.533	Very large
CRO	− 8.69 (52.1)	< 0.001	− 2.087	Very large
Translational study	− 10.20 (462.4)	< 0.001	− 0.898	Large
Central process	− 14.13 (20.5)	< 0.001	− 3.311	Extremely large*

CRO, Contract research organization; *Note: the sample size for the “No” CP is extremely small (n = 2), which may inflate the effect size. A non-parametric alternative (Cliff’s delta = 0.932) confirmed a very large effect.

A strong positive association was observed between OPAL scores and the number of assigned research personnel per study (ρ = 0.76, *p* < 0.001). Role-specific allocation was examined in relation to trial complexity as defined by OPAL_Total. A strong positive correlation was found between OPAL scores and the number of assigned research nurses (ρ = 0.74, *p* < 0.001), as well as data managers (ρ = 0.67, *p* < 0.001). Interestingly, CSC allocation showed a weak but significant negative association (ρ = − 0.17, *p* = 0.0001).

### Sensitivity Analysis

To assess the robustness of the discriminant validity findings, a sensitivity analysis was conducted using the OPAL score alone, excluding the optional components (i.e., study duration, sponsor/CRO involvement, and study type complexity). Kruskal–Wallis tests confirmed that OPAL remained significantly associated with key structural variables: study type (*χ*^2^_(13)_ = 334.29, *p* < 0.001), clinical setting (*χ*^2^_(14)_ = 200.93, *p* < 0.001), study duration (*χ*^2^_(5)_ = 31.11, *p* < 0.001), and enrollment size (*χ*^2^_(7)_ = 71.41, *p* < 0.001).

### Logical Consistency

**Fig. 2:**
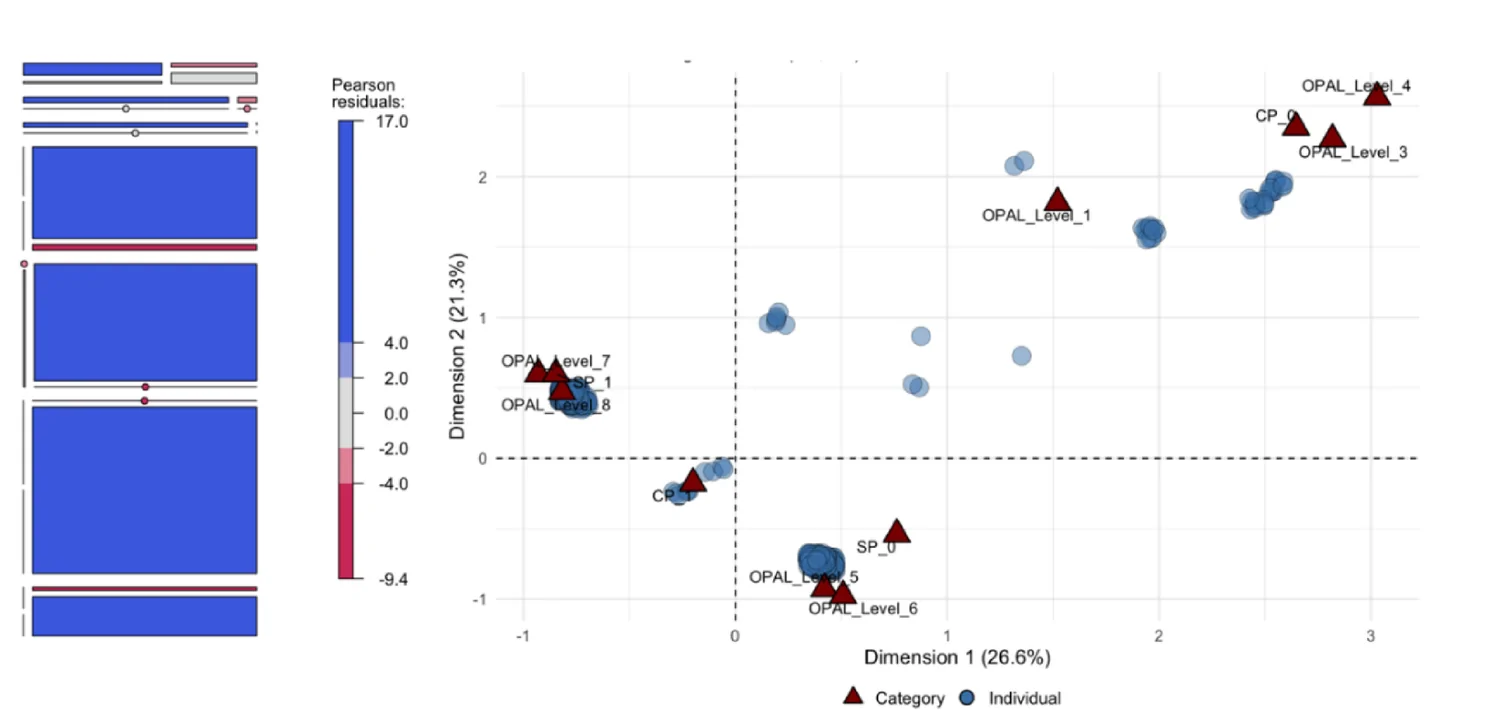
Associations between OPAL levels and key design features: centralized processes and translational focus. Exploratory association between OPAL levels and study design features (Centralized Processes [CP] and Translational Studies [Study Purpose; SP]). **Left**: Mosaic plot showing the distribution of OPAL levels by CP and SP. The color gradient represents Pearson residuals from the chi-squared test; strong blue or red areas indicate cells with significantly higher or lower counts than expected under independence (*p* < 0.001). **Right**: Multiple Correspondence Analysis (MCA) biplot illustrating the relationship between OPAL levels and the design features CP and SP. Triangles represent categorical variable levels (e.g., OPAL Levels, “CP_1_”, “SP_0_”), while jittered circles represent individual studies. Proximity between categories suggests association; for instance, OPAL levels 3–4 cluster near CP_0_ (no centralized processes), while OPAL levels 7–8 align with SP_1_ (translational studies)

We examined the internal consistency of the OPAL classification against its defining rule-set, including treatment type, centralized processes, and translational design. As expected, all studies classified as levels 1–3 were non-interventional, while levels 4–8 included only interventional studies. Furthermore, the distribution of OPAL levels across combinations of CPs and translational (SP) design confirmed the logical mapping: studies with both CP and SP were assigned to level 7, those with one or the other to levels 5–6, and those with neither to levels 4 or below (Fig. 2). No inconsistencies were observed. To evaluate the internal consistency of the OPAL classification system, we systematically verified the alignment between assigned OPAL levels and their defining rules. Specifically, we assessed whether: (i) non-interventional studies were confined to OPAL levels 1–3; (ii) interventional drug trials (rx) were assigned levels 4–8; (iii) Phase I studies were exclusively classified as OPAL 8; (iv) levels 5–7 matched the presence of CPs and/or translational design and (v) no study with complete information remained unclassified. The analysis revealed no inconsistencies (0 cases across all checks), supporting a logically coherent rule-based system with full alignment between input conditions and assigned OPAL levels.

## Discussion

**Fig. 3:**
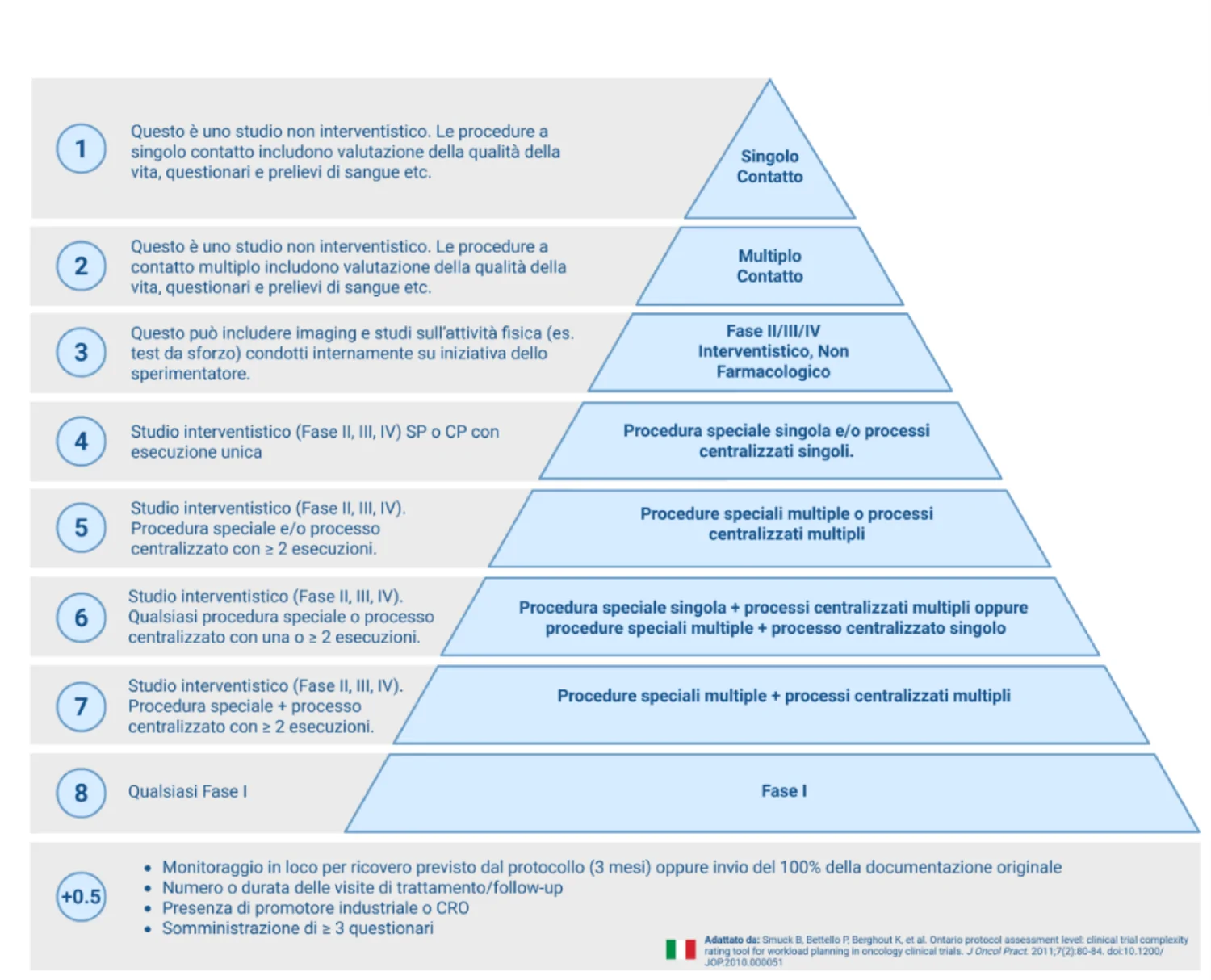
The Italian adaptation of the Ontario Protocol Assessment Level (IOPAL)

This study represents the first formal validation of the OPAL scoring system in the Italian clinical research context. The results demonstrate that OPAL is reliable tool with discriminant capacity for assessing clinical trial complexity and workload, with implications for research staffing, trial feasibility assessment, and operational planning. The high inter-rater reliability (ICC = 0.93) indicates strong consistency between independent raters, confirming that the OPAL classification can be applied reproducibly by trained professionals with minimal variation. This aligns with previous findings by Smuck et al., who originally introduced OPAL as a structured system to support resource allocation based on protocol demands [12]. High reliability is a crucial requirement for any tool intended for operational use in complex, multi-site environments (Fig. 3).

Beyond reliability, OPAL showed strong discriminant validity, with scores varying significantly across core trial characteristics such as study type, clinical specialty, sample size, and protocol duration. These differences were not only statistically significant but also substantial in practical terms, as reflected by large effect sizes, especially for study type. Such sensitivity indicates that OPAL effectively captures meaningful variation in workload burden associated with structural and procedural trial features. This aligns with previous evidence identifying trial phase, interventional design, and centralized oversight as key determinants of workload variability in both academic and industry-sponsored research [21–23].

The ecological validity of OPAL was further supported by its association with observed real-world staffing allocation patterns. Higher OPAL scores corresponded to a greater number of research personnel actually assigned to trials, suggesting coherence between the model’s complexity ratings and current operational practice. These data refer to assigned resources and should not be interpreted as a direct estimate of the optimal resources required by trial complexity. At the same time, the inverse relationship observed between trial complexity and CSC staffing highlights a potential mismatch between workload demands and formal role recognition. In the Italian context, where standardized definitions of research staff roles remain limited, this finding may reflect under-reporting or inconsistent attribution of research activities, rather than true reductions in staffing needs [15]. Such discrepancies underscore the importance of adopting standardized workload metrics to promote equitable task distribution and to prevent the concentration of excessive responsibilities on a limited number of professionals. Evidence increasingly links inadequate workload balance in high-complexity trials to elevated stress, reduced performance, and higher risk of burnout among clinical research staff [24, 25]. In this regard, OPAL may serve not only as a planning tool but also as a preventive mechanism to support workforce sustainability [4].

A particularly relevant contribution of this study concerns the impact of centralized processes and translational design features on trial complexity. Protocols involving centralized monitoring, randomization, and data management systems consistently received higher OPAL scores, confirming the substantial operational burden associated with sponsor-driven and multicenter infrastructures. The very large effect size observed for centralized processes highlights their dominant role in shaping workload, echoing previous findings on the intensification of trial demands linked to increasingly centralized governance models [26–28]. These results reinforce the need for feasibility frameworks that move beyond traditional indicators such as sample size or trial phase and incorporate organizational dimensions that critically affect day-to-day operations.

From an implementation perspective, OPAL offers a concrete response to organizational challenges frequently identified in clinical research. Prior studies have shown that factors such as unclear delegation, limited training, high staff turnover, and inadequate workload management contribute significantly to inefficiencies in trial conduct and delays in study timelines [29]. By providing a standardized and scalable method to quantify workload before trial activation, OPAL can support more informed feasibility assessments and facilitate alignment between protocol demands and available resources. In practice, OPAL scores could be integrated into pre-activation meetings and trial start-up checklists, informing staffing decisions, risk stratification, and negotiations with sponsors. Embedding such metrics in early planning phases may foster shared expectations among stakeholders and enhance organizational preparedness.

The potential utility of OPAL is further strengthened when considered alongside emerging operational roles, such as trial facilitators or start-up coordinators, whose function is to support sites during critical phases of trial initiation and enrolment. Recent evidence suggests that these roles can improve coordination and mitigate bottlenecks, particularly in high-complexity settings [30]. Systematic use of OPAL during feasibility assessments could help identify protocols that require enhanced support, guiding the strategic deployment of specialized personnel.

From a methodological standpoint, the robustness of OPAL was confirmed by sensitivity analyses, which showed that its core levels retained strong associations with structural trial features even in the absence of optional modifiers. This characteristic enhances the model’s applicability in contexts where complete protocol information may not be available, such as retrospective evaluations or administrative benchmarking exercises. Moreover, the internal consistency of OPAL’s rule-based framework was supported by the absence of classification inconsistencies: interventional studies were systematically assigned to higher complexity levels, whereas non-interventional studies remained confined to lower tiers. This internal coherence is a prerequisite for any tool intended for automated or semi-automated application in workload assessment systems.

### Limitations

Some limitations should be noted. The dataset was primarily composed of interventional and industry-sponsored trials, which may have limited its generalizability to academic or observational studies. Although this reflects current trends in Italy [31], future research should test OPAL in early-phase or investigator-initiated contexts. The minimal number of trials without centralized processes may have inflated effect sizes, despite robustness checks using Cliff’s delta. Workload was also not assessed at the individual staff level. Compared to national tools such as the IWAT and the NTRCT-AT, which focus respectively on CSC and CRN workload, OPAL offers a more holistic and scalable framework. Its tiered scoring system captures both protocol- and patient-level complexity, and its prior international validation reinforces its potential for multicenter coordination and benchmarking [13]. However, OPAL does not currently account for contextual variables such as institutional infrastructure, funding, or staff experience—all of which may influence perceived workload [4]. Integrating OPAL with broader workload and performance indicators could provide a more comprehensive view of trial feasibility [32].

Future research should explore the longitudinal implementation of OPAL in workload forecasting and workforce planning, as well as its validation across different therapeutic areas and European clinical research contexts. Particular attention should be paid to the heterogeneous Italian institutional landscape, including research settings with different governance models, staffing configurations, availability of dedicated research personnel, and levels of integration between clinical, pharmacy, laboratory, and administrative functions. Such studies would help clarify how contextual and organizational factors influence OPAL scoring, workload interpretation, and its practical use in feasibility assessment and resource planning. Further developments could also include the digital integration of OPAL within clinical trial management systems, enabling real-time complexity scoring and more dynamic allocation of research resources.

In conclusion, this study establishes OPAL as a reliable and discriminant tool for assessing clinical trial complexity in the Italian setting. By offering a structured and scalable method to quantify workload, OPAL supports evidence-based feasibility assessments, staffing decisions, and organizational planning in line with contemporary regulatory requirements. Its strong reliability and internal coherence make it suitable for application across diverse research environments. Integrating OPAL into feasibility meetings and trial start-up workflows may enhance transparency, resource alignment, and the long-term sustainability of clinical research infrastructure.

## Data Availability

The data presented in this study are available on request from the corresponding author.
